# Presbyopia developed earlier during the COVID-19 pandemic

**DOI:** 10.1371/journal.pone.0259142

**Published:** 2021-11-11

**Authors:** Kazuno Negishi, Masahiko Ayaki

**Affiliations:** 1 Department of Ophthalmology, Keio University School of Medicine, Tokyo, Japan; 2 Otake Clinic Moon View Eye Center, Kanagawa, Japan; National Taiwan University Hospital, TAIWAN

## Abstract

**Purpose:**

The aim of this cohort study was to evaluate the development and progression of presbyopia and the status of dry eye-related symptoms from 2017 to 2020, to assess the impact of the COVID-19 pandemic.

**Methods:**

Near add power at 30 cm was measured in 339 participants aged between 40 and 55 from 2017 to 2021 at Japanese eye clinics. Regression analysis of near add power and age was analyzed to compare 2017 with later years up to the pandemic. The prevalence of dry eye-related signs and six common symptoms were compared.

**Results:**

The number and mean age (y) of participants were 183 (48.6±4.1) in 2017, 46 (51.3±7.5) in 2019, and 110 (49.2±3.7) in 2020–21, respectively. The mean progression rate of near add power (D/y) was 0.13 for 2017, 0.09 for 2019 (P = 0.028, vs 2017), and 0.08 for 2020–21 (P<0.001, vs 2017). The slope (rate of presbyopia progression) became flatter from 2017 to 2021 and the estimated near add power at the age of 40 increased from 2017 to 2020–2021, implicating presbyopia developed earlier and worsened during the study period. The 2017 values were comparable with previous studies described in 1922 and 2019. The standardized correlation coefficient between age and near add power was 0.816 for 2017, 0.671 for 2019 (P = 0.084, vs 2017), and 0.572 for 2020–21 (P<0.001, vs 2017). Multiple regression analysis revealed age and COVID-19 pandemic were significantly correlated with near add power. The prevalence of dryness irritation, and pain was greater in 2020–21 than in 2017 with no difference in the prevalence of eye fatigue, blurring, and photophobia. There was no difference in the prevalence of short tear break-up time and positive corneal staining among 2017, 2019 and 2020–21.

**Conclusion:**

Estimated presbyopia developed earlier and progressed slower from 2017 to 2021, the COVID-19 pandemic. Stress and rapid digitalization related to strict infection control and quarantine might be contributing factors.

## Introduction

Since the COVID-19 pandemic was declared by the World Health Organization, drastic changes have developed worldwide in lifestyle, diet, exercise, and mental health [1] and numerous health problems have been documented. Ocular complications of COVID-19 and the pandemic include conjunctivitis, mask-associated dry eye and myopia progression [2–6].

Infection control, quarantine and working from home have been implemented in Japan, which, according to a national survey, has led to an increase in screen time and working at near distance in 2020 [7, 8]. Subsequently, ophthalmologists often encounter patients suffering from digital eye strain (DES). Drastic changes in work and life have led to tremendous stress and contributed to mental disorder in all generations [9–12]. There have also been discussions on the association between social media use and psychiatric disorders among the general public [10].

Numerous studies have been conducted in school children, university students and young adults to assess DES and report on subjective symptoms [12–16]. For example, sixteen symptoms relating to DES (headache, eye pain, heavy eyelids, redness of eyes, watering of eyes, burning sensation, dryness of eyes, increased sensitivity to light, itching, excessive blinking, difficulty in focusing printed text, blurring of vision, feeling that sight is worsening, feeling of a foreign body or grittiness of eyelids, double vision, colored rings around bright objects) were included in a questionnaire to respondents with a mean age of 27.4 years [12]. However, the status of middle-adulthood has not been investigated and real examination data has not been sufficiently documented.

Our clinical observation was that middle-aged patients suffered DES more seriously during quarantine due to the COVID-19 pandemic. Presbyopia and dry eye are serious health problems for the elder population. Presbyopia progresses as a consequence of aging and is associated with happiness and sleep [17, 18]. Dry eye is a common disease in middle adulthood and worsens with stress [19]. We hypothesized increased screen time and stress that occurred during quarantine may worsen presbyopia and dry eye in middle-adulthood. However, to the best of our knowledge, no study has investigated the status of presbyopia and dry eye during the pandemic to date. The aim of this study was to evaluate near add power and ocular surface signs and symptoms from 2017 to 2020 to explore how COVID-19 has affected accommodation and dry eye.

## Materials and methods

### Study design and participants

This study was a clinic-based, retrospective, cross-sectional study involving healthy subjects attending Tsukuba Central Hospital from January 2017 to July 2021. The Institutional Review Board and Ethics Committee of the Tsukuba Central Hospital approved this study (approved on December 12, 2014, permission number 141201). The Institutional Review Board and Ethics Committee of Kanagawa Medical Association (approved on November 12, 2018, permission number krec2059006) approved this study and participants were recruited from January 2019 to July 2021 at Otake Clinic Moon View Eye Center. This study was carried out in accordance with the Declaration of Helsinki. The need for consent was waived by the Institutional Review Board. Consecutive patients were analyzed during the study period. This clinical study was a retrospective chart review since examinations are routinely performed for patients older than 39 years in participating institutions. Every patient completed a health check sheet that included medical and family history before ocular examinations. The data from 2018 is lacking due to transfer of the investigator (MA). The Institutional Review Board and Ethics Committee of Keio University School of Medicine approved this study (approval date: June 28, 2021; approval number 20210080) to permit authorship for authors (KN and MA) who are appointed at the Keio University School of Medicine.

The all the data collected in this study, including the patient interviews, were collected as part of routine standard-of-care. Authors had access to information that could identify individual participants during or after data collection.

### Inclusion and exclusion criteria

Participants aged 40 to 55 years visiting for contact lens prescription with bilateral phakic eyes and best-corrected visual acuity above 20/30 were included in the study. Individuals were excluded if they had glaucoma, vitreoretinal disease, any ocular surgery in the previous month, acute ocular disease in the previous two weeks, or eyedrops potentially affecting accommodation (pinorexine [20] and cyanocobalamin).

### Patient interviews for dry eye-related symptoms

Patients were asked questions to determine the presence or absence of six common dry eye-related symptoms, namely dryness, irritation, pain, eye fatigue, blurring and photophobia. These questions were the six most prevalent symptoms of dry eye patients who had visited the Dry Eye Clinic in the Department of Ophthalmology at Keio University Hospital in 2014.

### Ophthalmological examinations

All patients were examined by board-certified ophthalmologists. Subjects with major age-related eye diseases, including cataract, glaucoma, and macular diseases were excluded. Ophthalmological evaluation consisted of best-corrected visual acuity (Vision Chart, SSC-370^R^, Nidek Co., Ltd., Gamagori, Japan), autorefractometry (TonorefTM II, Nidek Co., Ltd., Aichi, Japan), slit-lamp biomicroscopy, funduscopy, and intraocular pressure measurements (TonorefTM II, Nidek Co., Ltd., Aichi, Japan). Examiner measured binocular near add power at a distance of 30 cm using a Bankoku near-acuity chart (Handaya Inc., Tokyo, Japan) or an automatic optometry system (AOS-700^R^; Nidek Co., Ltd., Gamagori, Japan) [21]. After determining the patient’s distance refractive correction, the minimal additional power required to achieve near acuity above 20/25 at 30 cm was measured in 0.25 D increments, and was recorded as near add power. Dry eye-related examinations were performed according to standard procedures [22, 23] and consisted of tear break-up time (BUT) and a corneal staining test. BUT was measured using a saline-soaked (with excess flicked off) strip of fluorescein filter paper (Ayumi Pharmaceutical, Tokyo, Japan) applied to the lower lid margin, viewed with a suitable light source and yellow filter. The BUT was defined as the time interval between the third blink and the appearance of the first dark spot in the cornea measured using a stopwatch, taking the mean of three measurements. A BUT measurement less than or equal to 5 seconds was determined as a short BUT. Corneal staining was used to detect corneal epitheliopathy. Examination rooms were kept at 21–24°C and 40–60% humidity as recommended by the Japanese Ministry of Health and Labor.

### Statistical analysis

The sample size was calculated with a 0.05 margin of error and 95% confidence interval. Effect size was derived from a measured value in the current study. An effect size of 0.735 was identified in near add power with an appropriate total sample size for comparison of 2017 and 2020–2021 being 86. Where appropriate, data are given as the mean ± SD. We analyzed the data from the right eye for TBUT and refraction. A regression line for each year was computed for age and near add power by the least-squares method. The difference in slope (rate of presbyopia progression) among three regression lines was analyzed by a *t*-test. To identify which parameters correlated with near add power, a multiple regression analysis was performed with near add power used as the dependent variable, while demographic (age and sex), spherical equivalent, calendar year, and the presence of the COVID-19 pandemic used as independent variables. Near add power from patients aged 40 to 69 years was retrieved. Preliminary results indicated no difference in regression lines in 56- to 69-year-old patients across the three study periods. As such, we analyzed a younger subset of participants aged 40 to 55 years. All analyses were performed using StatFlex^R^ (Atech, Osaka, Japan) with *P* < 0.05 considered significant.

## Results

**Fig 1:**
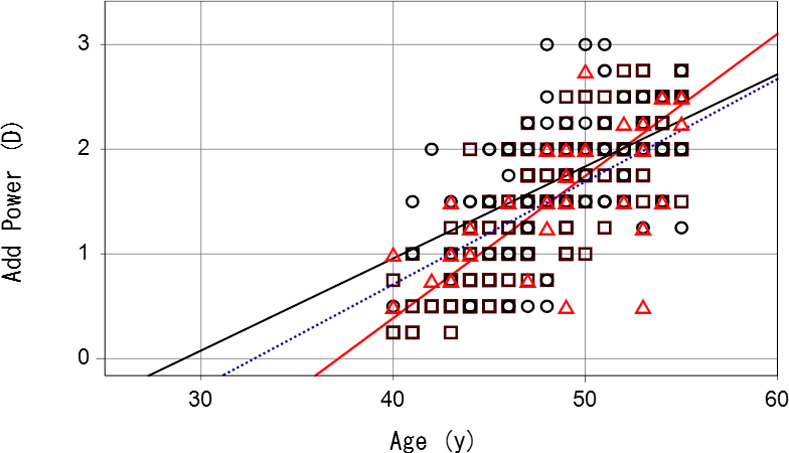
Regression analysis of age and near add power in 2017, 2019 and 2020–21. The slope (rate of presbyopia progression) became flatter from 2017 to 2021, implicating presbyopia developed earlier and progressed slower toward the pandemic in 2020–21. Red triangle symbol and red solid regression line for 2017, black square symbol and black dotted regression line for 2019, and black circle symbol and black solid regression line for 2020–21. Note many plots are overlapped and small number appears in the graph.

**Fig 2:**
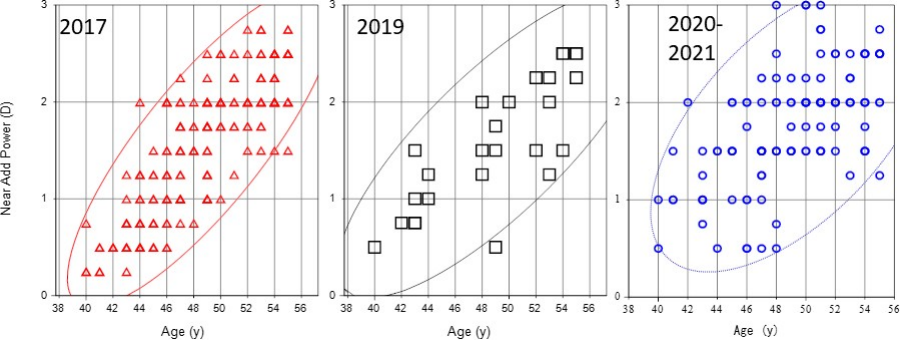
Scatter plots of near add power and age with a probability ellipse (confidence interval 95%) in 2017, 2019, and 2020–21. Correlation between age and near add power became weaker toward the pandemic 2020–21. The standardized correlation coefficient between age and near add power was 0.816 for 2017, 0.671 for 2019 (P = 0.084, t test, vs 2017), and 0.572 for 2020–21 (P<0.001, vs 2017). Note many plots are overlapped and small number appears in the graph.

Best corrected visual acuity was better than 20/25 in both eyes of all participants. The demographics and refractive and presbyopia status of participants in 2017, 2019 and 2020–21 are shown in Tables 1 and S1. Myopic error across the three study periods was greatest in 2017. Regression analysis of age and near add power (Fig 1) found the mean progression rate of near add power (D/y) significantly increased in 2019 (0.09, P = 0.028) and 2020–21 (0.08, P<0.001) compared with 2017 (0.13). The slope (rate of presbyopia progression) became flatter from 2017 to 2021 and the estimated near add power at the age of 40 increased from 2017 to 2020–2021, implicating presbyopia developed earlier and worsened during the study period. This indicates presbyopia developed earlier and progressed slower towards the pandemic in 2020–21. The standardized correlation coefficient between age and near add power was 0.816 for 2017, 0.671 for 2019 and 0.572 for 2020–21, with 2020–21 but not 2019 being significant different to 2017 (P<0.001 and P = 0.084, respectively; Fig 2).

**Table 1:** Refraction and presbyopia status.

Year	2017	2019	2020–21	P value	P value
				(2017 vs 2019)	(2017 vs 2020–21)
n	183	46	110		
Age	48.6±4.1	51.3±7.5	49.4±3.7	0.130	0.228
Sex (% male)	25.1	37.3	36.4	0.885	0.083
Spherical Equivalent (D)	-4.00±3.12	-2.58±3.15	-2.76±2.99	**0.002** *	**0.006** *
Astigmatism (D)	0.69±0.69	0.58±0.48	0.67±0.52	**0.027** *	0.827
Near add power (D)	1.55±0.68	1.84±0.79	1.77±0.61	<0.001*	<0.001*
Progression of presbyopia (D/y)	0.13	0.09	0.08	**0.028** *	**<0.001** *
Standardized correlation coefficient between age and near add power	0.816	0.671	0.572	0.084	**<0.001** *

*P<0.05, calculated by a t test except for sex, which was analyzed with chi square test.

A multiple regression analysis revealed age and the COVID-19 pandemic were significantly correlated with near add power (Table 2).

**Table 2:** Multiple regression analysis of near add power and possible confounding factors.

	ß	P value
Multiple regression analysis		
Age	0.754	<0.001*
^a^Sex	-0.005	0.854
Spherical equivalent	-0.023	0.432
^b^Calendar year	-0.017	0.843
^c^COVID-19 pandemic	0.181	0.041*
Stepwise regression analysis		
Age	0.754	<0.001*
COVID-19 pandemic	0.158	<0.001*

* P<0.05, Standardized partial regression coefficient.

^a^Men = 1, Women = 0.

^b^2017 = 0, 2019 = 1, 2020–21 = 2.

^c^2017 and 2019 = 0, 2020–2021 = 1.

The dry eye-related parameters of dryness irritation, and pain were significantly more prevalent in 2020–21 compared with 2017 (P = 0.002, 0.036, and P<0.001, respectively) (Table 3). No other dry eye-related symptoms were significantly different between two study periods.

**Table 3:** The prevalence of dry eye-related symptoms and corneal signs.

Year and prevalence (%)	2017	2019	2020–21	P value (2017 vs 2019)	P value (2017 vs 2020–21)
Eye fatigue	39.8	55.6	47.7	0.041*	0.216
Blurring	40.6	48.9	48.6	0.363	0.195
Photophobia	14.6	31.1	20.6	0.013*	0.175
Dryness	20.3	37.8	36.4	0.016*	**0.002** *
Irritation	14.6	26.7	24.3	0.067	0.036*
Pain	6.3	13.3	19.6	0.143	**<0.001** *
Short BUT	69.3	74.3	72.1	0.990	0.559
Positive corneal staining	23.8	14.7	15.4	0.603	0.095

*P<0.05, calculated by a chi square test.

BUT, tear break-up time.

## Discussion

The current study has revealed the COVID-19 pandemic may have modulated near add power in 40- to 55-year-olds. A multiple regression analysis of near add power and potential variables further confirmed an association between presbyopia and the COVID-19 pandemic. An increase of reported dryness, irritation, and pain may also implicate a presence of stressful ocular manifestations. The prevalence of short BUT and positive corneal staining did not change. These results suggest dry eye-related ocular symptoms may be due to psychologic issues related to the pandemic rather than physical deterioration. The regression line and values for 2017 were comparable with previous studies; with the onset (y) and progress of presbyopia (D/y) previously reported as 39.5 and 0.14 (Duane [24], 1922) and 38.7 and 0.13 (Ayaki [21], 2019), respectively. This further supports that presbyopia might be modulated during the pandemic. The current results may have sufficient generalizability since this is a multicenter study with minimized bias. Tonic pupil and some other ocular signs have been suggested as neuro-ophthalmological manifestations of COVID-19, however, these are not applicable to the present study [25, 26].

Some systemic illnesses and medications could affect the power of ciliary muscle and they should have been excluded from the study. In addition to aging as the greatest risk factor for presbyopia progression, myopia [27], hypermetropia [28], female sex [29], diabetes [30], alcohol intake [28], smoking [20], and pneuropsychiatric medications including sleep medicine, muscle relaxant, and sedative may be implicated. Pupillary diameter, corneal multifocality, and aberration may contribute to accommodation [31–34], with pupillary diameter most likely in 2020–21 [31]. A recent investigation using fMRI and pupillometry in medical residents found neural responsitivity of the noradrenergic locus coeruleus and associated pupil responses are related to changes in anxiety and depression in response to prolonged real-life stress [35]. Longitudinal survey results identified a worsening of psychological distress with COVID-19-related stressors [36]. Taken together, it could be hypothesized that early onset of presbyopia may be induced by decreased depth of focus evoked by an enlarged pupillary diameter that is associated with pandemic-related stress. The greater distribution of age-near add power in 2020 resulted in a greater standardized correlation coefficient, which may be due to individual variation in stress and digital work levels among participants. It is possible that the drastic and individual changes in lifestyle, work, screen time, and subsequent psychiatric distress deteriorated accommodation even in individuals who had not previously suffered presbyopia. Our results indicate that difficulty focusing may develop at presbyopic age due to COVID-19-related pandemic distress and as such, relevant optical and mental care should be considered. Our hypothesis of worsening of presbyopia in 2019 may be due to progressive digital eye strain and increasing mental stress during the study period, and the additional burden associated with the COVID-19 pandemic related to strict infection control and quarantine in 2020–2021.

Dry eye is closely associated with screen time and depression [37, 38], and both deteriorated during the pandemic. Preliminary results from examinations and surveys of eye clinic patients clearly indicated a decrease in lacrimal function and increase in the prevalence of dry eye-related symptoms occurred with the onset of the pandemic. Distress during the pandemic is more serious in the younger population and therefore a significant difference was not found in the present cohort.

The present study has several limitations. We must acknowledge the limitation of selection bias, as this study is clinic-based and people with any concerns may visit eye clinic. It is reasonable to assume that participants were from more-affected populations and experienced certain problems. While the difference in distribution and regression lines for 2019 and 2020–21 were statistically significant, sample size might be still insufficient and as such, the present results should be confirmed in a larger study. Participants in 2017 were myopic [27] and young compared with those in 2019 and 2020–21, as such, the near add power may possibly be affected by the refractive status and age. However, the regression line of 2017 data was comparable with two large previous studies [21, 24]. Furthermore, the difference between 2017 and the other periods may be due to stress and digital eye strain in 2019 and additional burden associated with the COVID-19 pandemic in 2020–21, rather than the effect of myopia and age. Pupillary diameter was not measured and this is a serious limitation. Screen time and use of digital devices should have been surveyed to determine DES.

## Supporting information

S1 Table The raw data of the subjects. (XLSX)

S1 File (DOCX)

## Abbreviations

DES: digital eye strain
BUT: tear break-up time
fMRI: functional magnetic resonance imaging
